# Care of the Teeth

**Published:** 1870-11

**Authors:** 


### CARE OF THE TEETH.

The best and safest tooth-wash in the world is tepid water. There is not a tooth powder in existence, nor a tooth-wash, that does not inflict a physical injury to the teeth, and promote their decay. Each dentist has a powder of his own, which he sells at a thousand per cent. profit, which he may honestly imagine will do a positive good without any injury whatever; but he is mistaken. The teeth were never intended to be pearly white. Every intelligent dentist knows that the whiter the teeth are, the sooner and more certain they will decay; he also knows that those teeth are the soundest, last the longest, and are the most useful, which have a yellowish tint; then why provide powders to take off this yellowish surface.

It is useful to wash the teeth once a week with white soap, making the mouth as full as possible with "lather," so as to be close to every particle of every tooth for a few minutes, because the tartar on the teeth is the product of a living thing, which is instantly killed with soap-suds. A few persons have another living thing about the teeth not affected by soap, but is instantly killed with salt; hence each person is advised to wash the teeth with white soap once a week, and once a week also with salt.

The teeth should be washed with a stiff brush every morning on rising, by dipping it once in water and rubbing the teeth slowly front and rear from side to side, and finally twisting the brush so that each bristle shall act as a tooth pick at the joinings of the teeth, so as the more thoroughly to dislodge anything which might remain in the hollows between the ridges. The water in the brush combines with the saliva of the mouth, and by its great softness makes one of the best solvents in nature for any extraneous substances about the teeth. The teeth should be brushed immediately after each meal with a soft, old brush, with plenty of water, twisting it up and down as before. After each washing, the brush should be placed far back on the tongue and turned from side to side, so as to clear off the tongue ; this does much towards freeing the teeth from the odor of the last thing eaten.

If persons would brush their teeth well immediately after the last meal of the day, instead of putting it off until bed-time, the teeth would be clean for three or four or five hours more in the twenty-four, which is not a slight advantage.

It is unnatural, absurd, and unphilosophical to have the teeth separated with a saw, or frequent picking, or by threads.

